# S100A4 overexpression proves to be independent marker for breast cancer progression

**DOI:** 10.1186/1475-2867-8-12

**Published:** 2008-09-05

**Authors:** Nawfal I Ismail, Gurjeet Kaur, Hasnah Hashim, Mohammed S Hassan

**Affiliations:** 1 Advanced Medical and Dental Institute (AMDI), Universiti Sains Malaysia (USM), Penang, Malaysia

## Abstract

**Background:**

Breast cancer is the most common cancer and cause of deaths in women around the world. Oncogene amplification usually occurs late in tumor progression and correlates well with aggressiveness of tumor. In fact the function of the S100A4 protein and its role in metastasis is unclear at present. The purpose of the study was to determine the expression of S100A4 protein in the invasion status and metastatic potential of breast cancer by using tissue microarray and to determine its role in breast cancer based on the expression of S100A4 gene product.

**Methods:**

S100A4 protein expression was examined by immunohistochemistry (IHC) using commercially available tissue microarray containing malignant and normal breast tissue cores from 216 patients.

**Results:**

S100A4 was absent in normal breast tissues while positive in 45.1% of infiltrating ductal carcinoma (IDC) node negative and 48.8% of infiltrating lobular carcinoma node negative. In paired samples, S100A4 protein was expressed in 13.5% of IDC node positive cases and 35.1% of matched lymph node metastasis.

**Conclusion:**

S100A4 protein expression appears widely expressed in early and advanced breast cancer stages compared with normal breast. Our study suggests S100A4 may play a role in breast cancer progression and may prove to be an independent marker of breast cancer which appears to be down regulated in more advanced stages of breast cancer.

## Background

Breast cancer is still the most common public health problem in women worldwide [1]. Cancer cells may invade the surrounding by tissue remodeling and angiogenesis [2-7]. They may spread through the bloodstream and lymphatic system to other parts of the body [2-7]. The majority of invasive breast cancer from the epithelium of lobules and ducts of the glands [8-21]. Metastasis is considered as the spreading of tumor cells from the primary neoplasm to distant sites [22,23]. In spite of significant advancement in early diagnosis, surgical intervention as well as local and systemic adjuvant therapies, the majority of cancer deaths are attributable to metastasis that are resistant to available therapies [22].

The metastasis process is not a random process but consist of a complex series of linked and interrelated steps involving multiple host-tumor interaction [24]. Many proteins including proteases, adhesion molecules, angiogenesis, and growth factor are involved in metastasis [25]. Therefore, understanding the gene and protein expression changes in metastatic cancer cells and nearby cells of the microenvironment may aid in early diagnosis and therapeutic intervention. During the last decade, considerable progress has been made in understanding these changes at the molecular level. In fact most deaths of women with breast cancer arise not as a result of primary tumor but from its metastatic spread to distant sites in the body [26,27]. Once spread and secondary masses are formed, breast cancers are usually incurable [28]. Yet a sensitive and reliable method for early metastasis in breast cancer is still not available.

Breast cancer is considered to be a systemic disease this would mean that the most breast carcinoma metastasize before diagnosis of the primary lesion [29]. Therefore, early detection of metastasized lesion and identification of more effective therapeutic modalities for metastatic disease are necessities if the prognosis for patients with advanced breast cancer is to improve.

The S100 gene family located on chromosome 1q21, comprises more than 20 members whose protein sequences encompass at least one EF-hand Ca++ binding motif [30,31]. S100 is a 21 Kd highly acidic and water soluble calcium binding protein [32]. S100A4 gene occurs in cluster of 13 S100 genes on chromosome 1 [33], which are also often amplified in cancer of the breast and which contains jumping elements [34]. The expression of individual family members is not permanent for all tissues and appears to be an element of tissue specific expression. S100A4 is composed of an alpha and beta chain with molecular weight of 10 – 12 Kd [35]. It is a small molecule and can pass through the nuclear pores without any active transport mechanism being involved. S100A4 binds and inhibits phosphorylation of the p53 C-terminal peptide by protein kinase C. The tumor suppressor protein p53 has also been identified as an S100A4 interacting protein and may provide a link between S100A4 and apoptosis [36]. p53 is a critical tumor suppressor that is involved in most if not all tumorigenesis. Almost 30–50% of breast cancers contain a p53 mutation [37]. Other reported S100A4 interacting proteins include tropomyosin, methionine aminopeptidase, and CCN3 (cysteine-rich 61/connective tissue growth factor/nephroblastoma overexpressed) [38-40].

Studies to determine the mechanistic basis for S100A4 function have shown a potential role for S100A4 in several different facts of tumor progression including motility, invasion, and apoptosis [[36,41], and [42]]. It has also been reported that extracelluar secreted S100A4 can affect cell differentiation and migration [42-44]. Elevated levels of immunocytochemically detected S100A4 are associated with the more malignant carcinomatous regions of the primary tumor and with liver metastasis [45]. An increase in S100A4 protein expression has been correlated with a worse prognosis for patients with different types of cancer including colorectal, gallbladder, bladder, esophageal, breast, and non small lung cancer [46-52]. The main purpose of the study was to find the expression pattern of S100A4 proteins in different types of breast cancer with or without lymph node involvement and to determine its role in breast cancer using tissue microarray.

## Methods

Formalin-fixed paraffin-embedded tissue microarrays from 188 lymph node negative breast cancer patients, 50 breast cancer patients with lymph node metastasis (50 malignant tissues and 50 matched lymph node tissue cores with metastasis) and 8 normal breast tissue cores were analyzed by immunohistochemistry for the expression S100A4 protein. Included in this study were patients with infiltrating ductal carcinoma, infiltrating lobular carcinoma, normal breast tissue and lymph node metastasis. The final number was 122 tissue cores of node negative infiltrating ductal carcinoma, 41 node negative infiltrating lobular carcinoma, seven normal breast tissue, 40 node positive (38 infiltrating ductal carcinoma, 2 infiltrating lobular carcinoma) and 46 tissue cores of lymph node metastasis. We did not evaluate infiltrating lobular carcinoma node positive because of the low sample size (2 cases). Forty six tissue cores were excluded from statistics because very little cancer cells or no breast cancer tissue were seen. The total number after exclusion was 254 tissue cores (37 matched tissues) of 216 patients.

### Immunohistochemistry (IHC)

Rabbit Anti-Human polyclonal primary antibody against S100A4 protein (Code No. A5114, from DakoCytomation, Denmark) was used on deparaffinized tissue microarray slides (Cat. No. BR 2001, BR 1001 from Biomax, USA). A secondary detection system (DAKO Envision) enhanced with conjugated polymer was used to bind with the primary antibody. DAB chromogen was used for permanent color development and detection under microscope.

The percentage of carcinoma cells with cytoplasmic/membranous/nuclear staining was recorded on each specimen at 200× magnification, using light microscope. The expression of S100A4 was scored in all tumors as: positive ≥ 5% and negative < 5% stained cells. Also the intensity of staining was categorized into three groups: weak, moderate and strong. This was ascertained by a single qualified pathologist.

**Figure 1:**
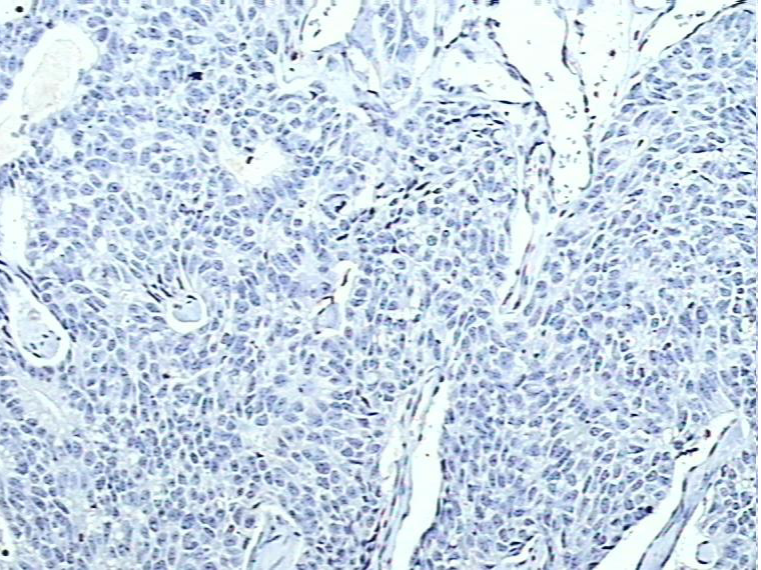
Negative control, S100A4 protein immunohistochemical staining in breast cancer tissue, showing absent staining (×200).

A tissue section of breast cancer was used as positive control for S100A4. Rabbit IgG isotype (Sigma-Aldrich, USA) was used instead of primary antibody in the immunohistochemical technique on a tissue section each of breast cancer and normal breast as negative control (Figure 1).

The tissue microarray slides were placed on hot plate at 60°C for 30 minutes. The slides were immersed in two changes of xylene. Slides were then immersed in 3 different concentrations of ethanol. Slides were rinsed with distilled water to remove ethanol. The slides were then placed in target retrieval solution EDTA buffer pH 9.0 (DAKO) and heated on microwave. Slides were allowed to cool at room temperature and rinsed with Tris Buffered saline (TBS) mixed with tween 20. Slides were covered with peroxidase blocking solution (DAKO), followed by rinsing with TBS buffer mixed with tween 20. Then 200 μl of primary antibody (dilution 1:200) was added on the tissue microarray slides, followed by rinsing with TBS mixed with tween 20. Two drops of DAKO Envision/HRP, Rabbit/Mouse (secondary antibody) were added on the slides, followed by rinsing with TBS mixed with tween 20. After that DAB substrate (DAKO) was added on section slides followed by rinsing, immersion into hematoxylin and 4 different concentrations of ethanol. After that slides were immersed in two changes of xylene and cover slipped. All incubation steps after heat induced epitope retrieval were carried at room temperature.

## Results

The S100A4 protein was expressed in the cell cytoplasm without evidence of nuclear staining.

There were a total of 122 cases of infiltrating ductal carcinoma node negative and 41 cases of infiltrating lobular carcinoma node negative. Infiltrating ductal carcinoma node positive consisted of 38 cases. Thirty seven cases had paired primary infiltrating ductal carcinoma tissue with its matched lymph node core. There were also 9 cases of unrelated lymph node cores containing metastatic deposits.

**Figure 2:**
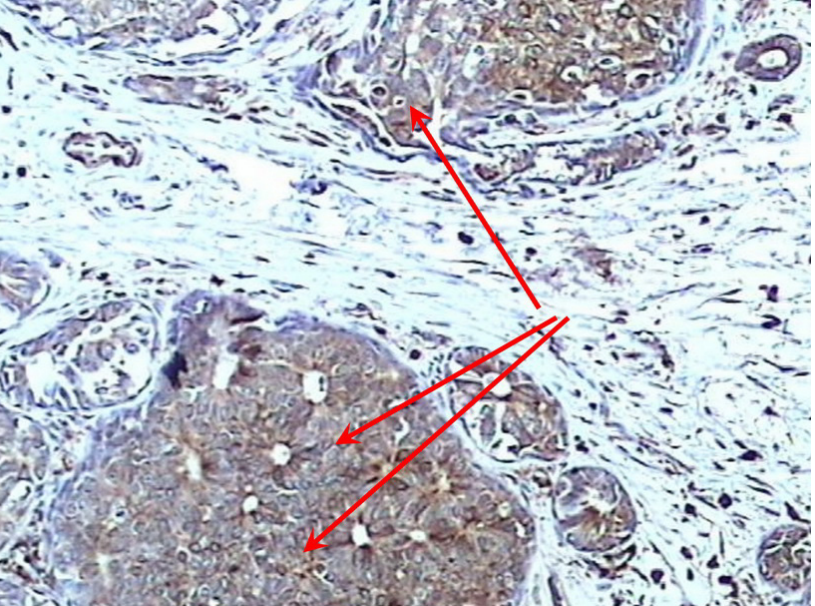
Positive expression of S100A4 within infiltrating ductal carcinoma node negative (×200).

**Figure 3:**
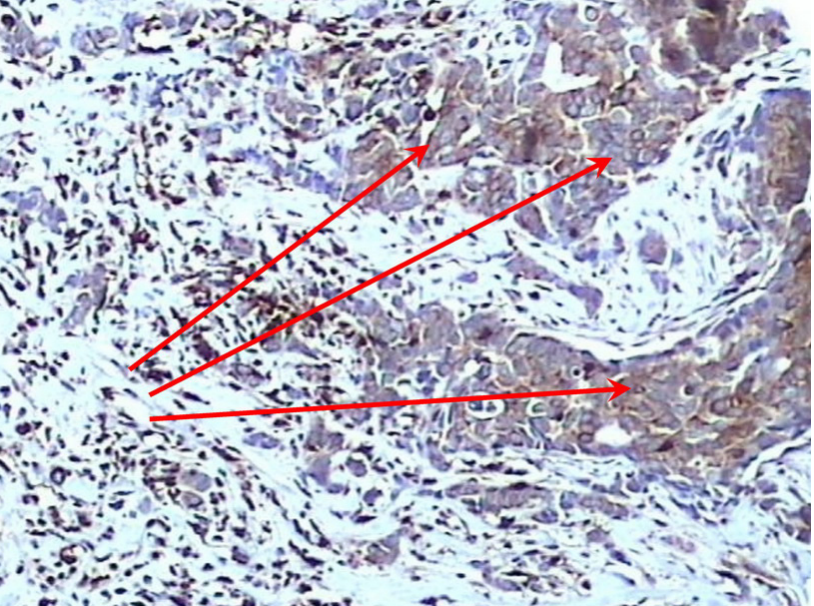
Positive expression of S100A4 within infiltrating lobular carcinoma node negative (×200).

**Figure 4:**
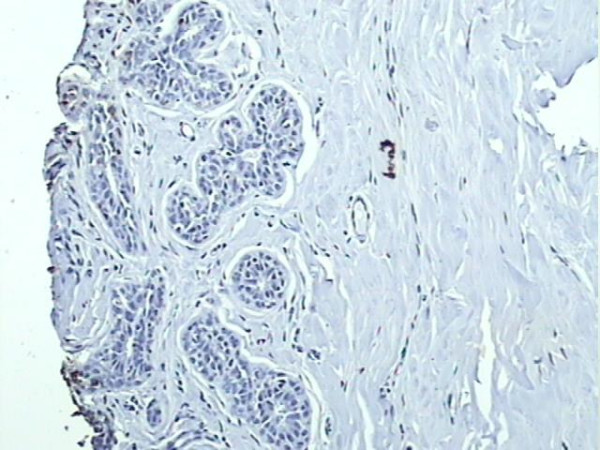
Negative expression of S100A4 protein within normal breast tissues (×200).

A positive expression of S100A4 was observed in 45.1% (55/122) cases of infiltrating ductal carcinoma node negative (Figure 2) and 48.8% (20/41) cases of infiltrating lobular carcinoma node negative (Figure 3). S100A4 staining was not observed in normal breast tissues (Figure 4).

**Figure 5:**
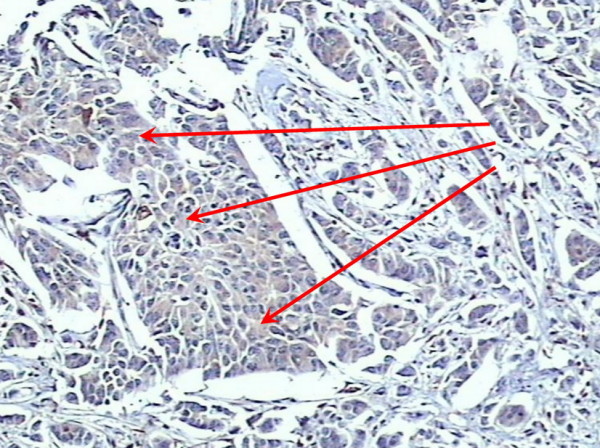
Positive expression of S100A4 within matched lymph node metastasis (×200).

Five of 37 (13.5%) cases in the paired samples (primary breast carcinoma and matched lymph nodes) showed presence of S100A4 protein in the primary site, while 13/37 (35.1%) cases showed S100A4 in lymph node (Figure 5).

It was observed that 12 of 17 (70.5%) paired cases (primary breast cancer with matched lymph node) showed expression of S100A4 in the lymph node metastasis with absent expression in the primary site. Four (23.5%) cases showed S100A4 expression in the primary breast carcinoma with absent expression in its matched lymph node metastasis. One case showed S100A4 expression in both sites.

## Discussion

Cancer is a disease or disorder characterized by uncontrolled growth (division) of the cells [53-55] and their ability to invade other organ or tissue, either by invasion or metastasis [54-56]. Although there are many types of cancer, all cancer types begin with uncontrolled growth of abnormal single cells in the body [55]. Oncogene amplification usually occurs late in tumor progression and correlates well with aggressiveness of tumor [57]. The function of the S100A4 protein and its role in metastasis is unclear at present. It has been reported that S100A4 may affect the function of cytoskeletal proteins including actin and non muscle myosin [58,59] so it is possible that S100A4 may regulate cell shape and or motility. It was reported that over expression of S100A4 protein is closely correlated with many functions for tumor aggressiveness, such as lymph node metastasis [60].

In the present study, S100A4 protein expression was examined by immunohistochemistry in infiltrating ductal, infiltrating lobular carcinoma and lymph node metastasis and their relation to tumor promotion and progression. Positive expression of S100A4 was observed in 45.1% of the infiltrating ductal carcinoma node negative cases, while in infiltrating lobular carcinoma with node negative, the expression of S100A4 protein was observed in 48.8%. This shows that S100A4 has a similar expression level in both infiltrating ductal carcinoma and infiltrating lobular carcinoma with node negative. S100A4 staining was not observed in normal breast tissues. S100A4 was expressed in a higher percentage in breast cancer tissue compared to normal tissue which showed direct correlation of S100A4 protein in infiltrating breast cancer node negative. This suggests that S100A4 is over expressed in infiltrating breast carcinoma compared to normal breast. Positive expression of S100A4 protein was observed only in 13.5 % of cases of infiltrating ductal carcinoma node positive while positive expression of S100A4 protein was observed in 35.1% of matched lymph node metastasis. These results showed there was a decrease in expression of S100A4 in infiltrating ductal carcinoma node positive (13.5%) compared with IDC node negative (45.1% positive staining), but interestingly there is an increase of expression of S100A4 protein at metastatic lymph node site. This suggests S100A4 may play a role in advanced breast cancer especially with lymph node metastasis. It was also interesting to note that the expression was seen in one site i.e. either primary tumor or its metastatic lymph node only.

The majority (70%, 12/17) of paired samples showed that when there was a positive expression of S100A4 protein in metastatic lymph node, there was associated negative expression in the primary tumor (infiltrating ductal carcinoma) of the same patient. One case showed positive expression in both primary tumor and its metastatic lymph node at the same time. This was comparable with another study which showed that S100A4 over expression directly correlated with tumor progression [60]. This study found that S100A4 was expressed in more cases of metastatic lymph node (35.1% cases) compared to matched infiltrating ductal carcinoma node positive (13.5% cases). These results show that there was a decrease expression of S100A4 protein in the primary tumor and increase expression in the metastatic lymph node for the same patient. This suggests that S100A4 is highly expressed in newly growing cancer either in the primary or metastatic sites. S100A4 protein was expressed in the cytoplasm of node negative IDC and ILC, whereas decreased in more advanced cancer (node positive). The reduction in S100A4 expression in the primary site may probably be related to cancer cells in the process of migration to distant sites.

## Conclusion

In conclusion S100A4 protein expression appears to be expressed widely in early and advanced stages of breast cancer compared with normal breast. This study indicates a complex role of S100A4 in breast cancer of different types and stages. The difference in the expression of S100A4 protein suggests it may be useful as an independent marker of breast cancer which appears to be down regulated in more advanced stages of breast cancer. However a larger study with more ILC and metastatic cases may clarify the role and function of S100A4 in breast cancer progression.

## Abbreviations

IHC: Immunohistochemistry; IDC: Infiltrating ductal carcinoma; ILC: Infiltrating lobular carcinoma.

## Authors' contributions

NII carried out Immunohistochemical part of the study and lab work, participated in drafting the manuscript. GK carried out the pathological part of the study, participated in drafting the manuscript. HH performed the statistical analysis. MSH initiated the project, participated in drafting the manuscript. All authors read and approved this manuscript.
